# Case report: Electrocardiographic (ECG) recording during the hanging process

**DOI:** 10.1007/s12024-024-00869-6

**Published:** 2024-08-12

**Authors:** E. M. Ungermann, M. Balikowski, J. Hemker, K. Feld

**Affiliations:** 1 Institute of Forensic and Legal Medicine, University Hospital Heidelberg, Vossstrasse 2, 69115 Heidelberg, Germany; 2 Zero Praxen Ludwigshafen, Erzbergerstraße 113, 67063 Ludwigshafen, Germany

**Keywords:** Postmortem ECG analysis, Strangling, Perimortal ECG changes, AV block

## Abstract

A 48-year-old woman was found hanged in the bathroom. She was wearing a Holter monitor, which was later analysed by a cardiologist. During autopsy, findings congruent with atypical hanging were collected. The ECG showed a 20 s asystole and four minutes later bradycardia, which progressed to a second-degree AV-block Mobitz I, then Mobitz II, then to a third-degree AV-block. Finally, only P waves could be observed, before heart action ceased. This is one of few cases reporting ECG-changes during hanging and might give further insight into the complex pathophysiology of this type of death.

## Introduction

The most common methods of suicide include hanging, firearm suicides, poisoning, drowning and jumping from heights [1]. While interruption of cerebral blood flow is thought to be the primary cause of death in hanging, the exact mechanism is yet to be determined [2]. Hanging is classified into typical and atypical categories to distinguish between different pathophysiological mechanisms. According to Patscheider and Hartmann [3], atypical hanging is classified based on the placement of the knot, with one side of the ligature running over the mandible. This results in an intact or partially intact arterial blood flow of the brain with congestion of blood in the head due to venous blockage. Typical signs of atypical hanging include petechial haemorrhages above the hanging mark, cyanosis and blood stasis in the head [2]. In typical hanging, petechial hemorrhages of the face are usually absent [2].

Little is known about the pathophysiology of hanging in general [4] as well as the effect of cardioinhibitory reflexes and mechanisms on death in hangings. An old textbook from 1895 [5] stipulated that applying pressure to the carotid artery and vagus nerve can stimulate the parasympathetic nervous system, thereby reducing blood pressure and heart rate. However, experiments conducted by Placzek in 1901 [6] demonstrated that direct pressure on the vagus nerve does not affect parasympathetic feedback loops, concluding that the observed effects were due to pressure on the carotid sinus. In contemporary literature, the influence of vagal reflex mechanisms on deaths in hanging is considered to be of minor significance [2, 7, 8].

## Case report

**Fig. 1:**
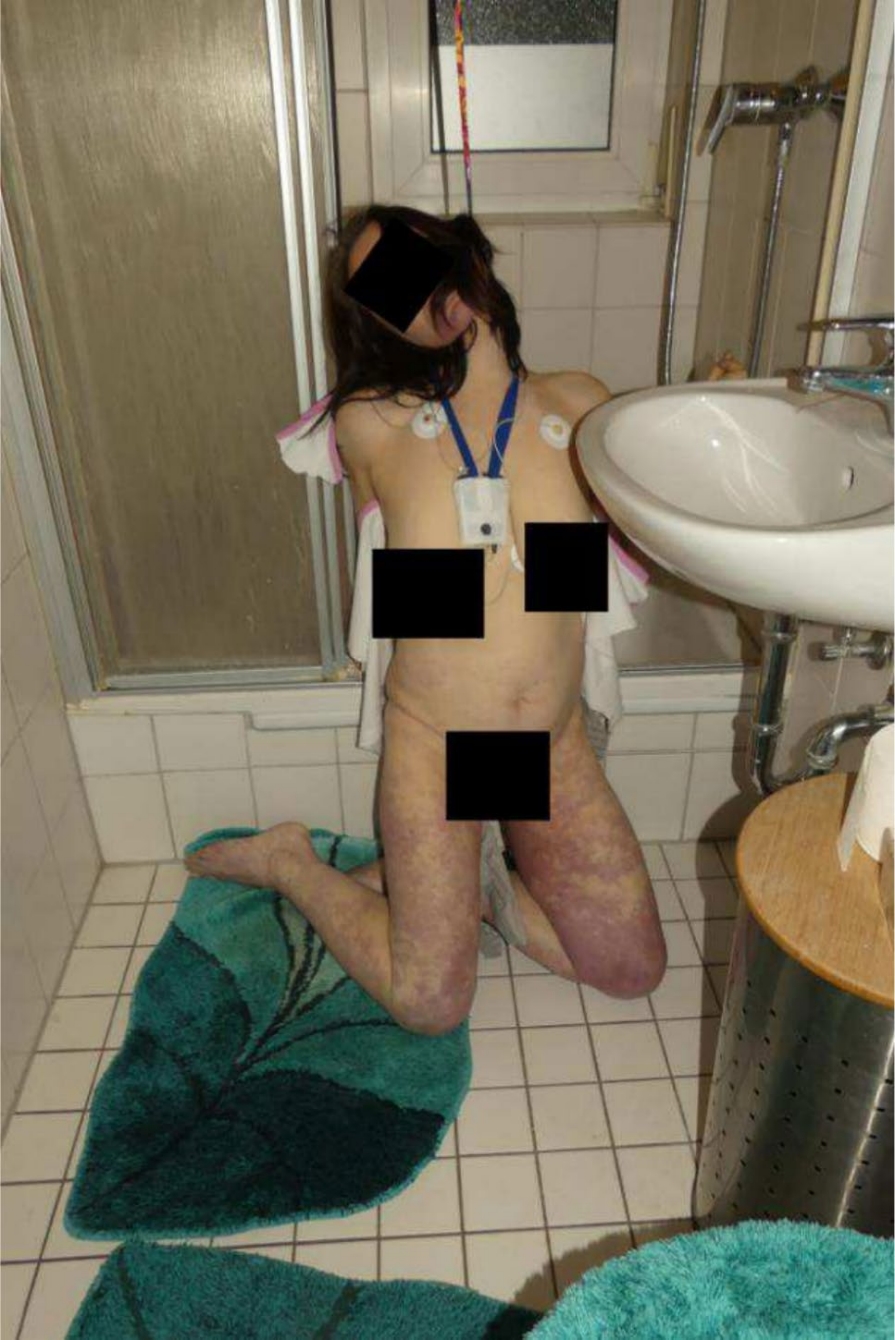
Initial placement of the body. The Holter monitor is still worn. Knot placement on the left side of the neck

A 48-year-old woman was found hanged in the bathroom. The body was found kneeling in front of the shower, the back turned towards the bathtub and partially suspended from a satin-like belt tied to the frame of the shower cubicle. The knot of the belt was located on the left side of the neck 1 m above floor level. During postmortem examination, a Holter monitor was found with electrodes placed below each collarbone (Fig. 1). According to the daughter, the Holter monitor was being used to evaluate chest pain.

The police found small quantities of marihuana in the living room and suspected its consumption prior to the death. Forensic experts were only involved after the body was brought in for autopsy; thus all mentioned circumstances are based on the photographs and reports by the police.

## Results

### Autopsy findings

**Fig. 2:**
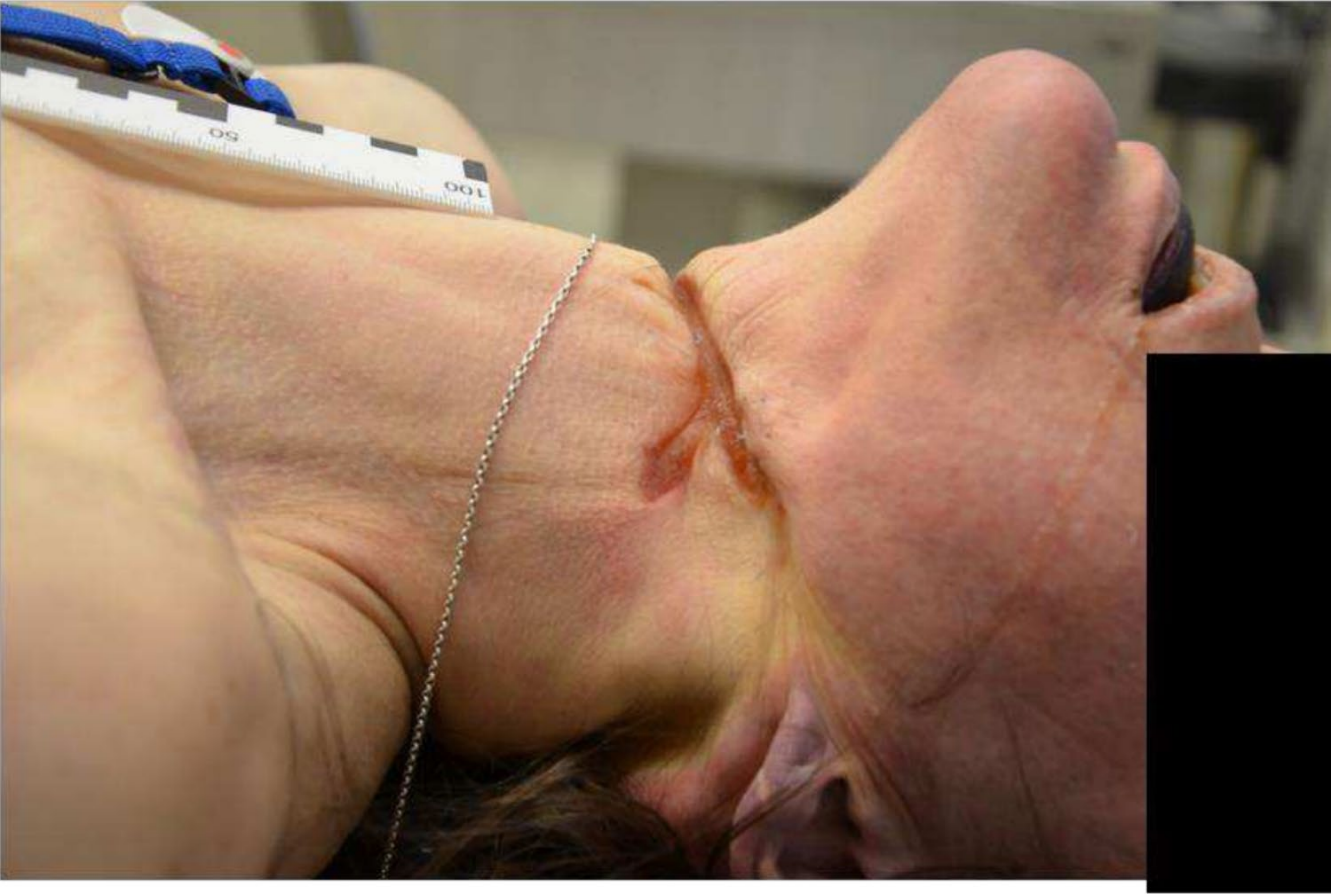
Hanging mark with the initial knot placement on the left side of the neck

According to the autopsy, the cause of death was determined as cerebral ischemia, secondary to hanging. The manner of death was declared non-natural (suicide). Several petechial haemorrhages were found on the facial skin, eyelids, conjunctives, oral vestibular mucosa, and in the skin behind the ears. The anal region was soiled with faeces. A hanging mark corresponding to the satin-like belt was noted (Fig. 2). The heart and carotid sinus showed no significant changes. Small haemorrhages at the insertions of the sternocleidomastoid muscles to the collarbone were observed. The hyoid bone and larynx were intact. No competing causes of death were identified.

Since the cause of death was conclusive, no further investigations were carried out, according to local guidelines. In preparation for publishing this case, a screening for alcohol and toxicologically relevant substances was conducted. At the time of analysis, no histology was available due to the statutory disposal of the biological material.

### Toxicological analyses

Alcohol analysis by gas chromatography–mass spectrometry (GC–MS) revealed a blood alcohol concentration of 1.44 ‰. No other toxicologically relevant substances, especially no THC, were found the blood or urine through general unknown screening.

### Holter monitor analysis

**Fig. 3:**
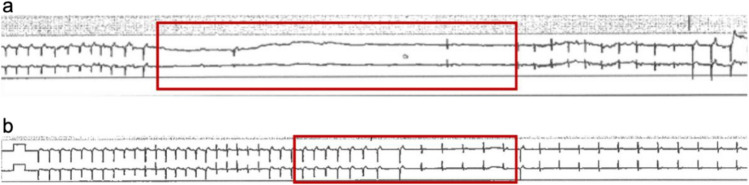
Asystole in ECG of 20.9 s (**a**) and sudden onset of bradycardia (**b**), the red square marks the ECG changes

The ECG recordings started at 13:10. At the beginning no abnormalities were recorded, and the mean heart rate was around 90 – 95 bpm. No arrhythmias were detected. A total of five supraventricular extrasystoles were recorded over a period of 8.5 h. An asystole of 20.9 s occurred at 21:41 (Fig. 3 a). At 21:45, a sudden bradycardia fragment can be observed, with the heart rate dropping to 50 bpm (Fig. 3 b). At 21:48, a second-degree atrioventricular (AV) block type Mobitz I (Wenckebach) occurred, and at 21:49 it progressed to a second-degree AV-block type Mobitz II, and further to a third-degree AV-block. At 21:49, the ECG recorded an asystole showing only p-waves. At 21:52 the p-waves ceased. In line with statutory requirements, the ECG was interpreted by a cardiologist.

## Discussion

To our knowledge, this is the second case report documenting ECG changes during hanging [9]. While changes in the ECG of hanging survivors, such as elongated QTc intervals and initial ventricular fibrillation, are widely described [10], evidence of cardiophysiological stigmata in cases of complete hanging remains scarce.

In the present case, there was an abrupt onset of bradycardia, followed by the progression of AV blocks. Although the other published case [9] did not discuss the ECG findings in detail, a photograph of the trace was taken. A cardiologist conducted a comparative interpretation of these two ECGs and found a similar electrophysiological picture. In our case, an asystole of about four minutes preceded the onset of bradycardia and the consecutive progression of AV blocks. In the other published case, a second-degree AV block with a conduction pattern of 2:1, then 3:1, and then 4:1 was observed. Subsequently, no QRS complexes were noted, and finally, the P-waves ceased as well.

Based on these observations, we propose that the abrupt onset of bradycardia marks the moment of hanging when the carotid sinus is compressed. This indicates that the pressure on the baroreceptors caused by the ligature may also play a role during hanging.

The reason for the initial asystole of about 20 s in the present case can only be hypothesized. The ECG showed no signs of disturbance, as would be expected in artifacts caused by seizures. One possible explanation is an initial attempt at hanging, where either the ligature broke or the attempt was abandoned. However, no morphological signs of a first attempt of hanging was found on the body, especially no second ligature mark.

Another possible explanation is that the initial asystole marks the point of hanging. The later noted bradycardia could be interpreted as a heart rhythm resulting from the dying of the heart itself, which is not directly linked to the cause of death. In the literature, the rhythm of the dying heart is described as different types of arrhythmias, with terminal irregular idioventricular rhythms and gradual slowing and disappearance of P waves [11]. These kinds of changes were not observed in our ECG. Instead, the final rhythm recorded showed only P waves. The other published case showed a similar pattern, with only p waves as the final rhythm before asystole.

Different modes and mechanisms of death in hanging are discussed in the literature [4]. In 1870, Ecker reported a hanging which resulted in an obstruction of the airways [12], later Langreuter identified four different mechanisms of death in 1886. These authors proposed that death by strangulation could result from compression of the airways, compression of the blood vessels supplying oxygen to the brain, compression of the vagal nerve, or injuries to the spine [13]. It is also possible that negative chronotrope and negative dromotrope effects on the heart due to compression of the nerves along the carotid arteries contribute to the cause of death [8].

Especially in atypical hangings, compression of venous vessels without complete compression of arterial vessels is observed, resulting in congestion and the occurrence of petechial hemorrhages above the strangulation level [14]. Death is typically attributed to brain hypoxia [2, 14]. One mechanism worth discussing is whether pressure on the carotid sinus induces a vagal reflex, thereby leading to bradycardia [7].

Based on experiments conducted by Brinkmann et. al. on dogs, [15] it has been determined that in atypical hangings, there is a drop in heart rate and blood pressure. Consciousness is typically lost after about 8 s, and breathing activity ceases. Asphyxia could be induced by clamping the endotracheal tube used for ventilation, resulting in corresponding changes in the ECG such as ventricular fibrillation, followed by progression to AV block and ultimately pulseless electrical activity [16]. While direct comparisons with humans can be challenging, similar results have been observed in cases of survived hanging, where ventricular fibrillation may occur initially [10].

In self-hanging experiments conducted by Minovici in 1904, loss of consciousness occurred after 8–9 s when the knot was located laterally on the neck [17].

In the present case, no ventricular fibrillation could be observed. This suggests that the observed changes are not consistent solely with those resulting from oxygen deprivation, thereby making the involvement of vagal reflex mechanisms even more likely. According to the literature, cessation of blood circulation in the brain, which is the predominant factor in hanging, leads to loss of consciousness within 6–10 s [2]. In contrast, experiments involving the cessation of blood flow to the brain in volunteers using mechanical cuffs over a period of 100 s did not report significant health effects [18].

While slowing of the heart can be observed in asphyxia [19], the abrupt onset of bradycardia in the current case needs to be discussed. Bradycardia occurred abruptly within seconds in the present case, suggesting that it was not solely due to cessation of brain circulation. Based on current evidence, it could be stipulated that there was a failure of the central regulatory mechanisms that control the heart and breathing. Lack of oxygen and brain congestion can lead to a Cushing reflex and subsequent increase in sympathetic output [20]. These effects contrast with the ECG recordings in the present case, which showed progressive AV block due to activation of the parasympathetic nervous system. Such activation of the parasympathetic nervous system can be induced by pressure on the carotid baroreceptors.

The effects of alcohol consumption on the ECG should also be discussed. While the recorded changes in the ECG are likely directly linked to the hanging and subsequent death, the presence of 1.44 ‰ of alcohol may have also contributed to these changes. Most ECG changes recorded during acute alcohol intoxication include atrial fibrillation or premature ventricular contractions, but in some cases, atrioventricular conduction disturbances could be observed. However, based on the balance of probabilities, the recorded ECG changes were more likely a result of increased vagal tone rather than the effects of alcohol itself [21].

Due to the small number of cases, with only two publications including the one we presented, it remains questionable whether our findings can be generalised to other similar cases. One opportunity for a larger dataset could arise from the increasing number of smartwatches with ECG recording capabilities, which may provide new insights into the cardiac pathophysiology of hanging.

## Key points

1. This case report documents ECG-findings in a case of atypical hanging.
2. This observation can give further insight into the pathomechanisms in hanging.
3. Inhibitory mechanisms might play a role in atypical hanging.
4. A 48-year-old woman was found hanged in the bathroom, while wearing a Holter monitor. The recorded electrocardiogram (ECG) was subsequently analyzed by a cardiologist. Autopsy findings indicated characteristics of atypical hanging. The ECG revealed a 20-s asystole, followed four minutes later by bradycardia that progressed to a second-degree atrioventricular (AV) block Mobitz I, then Mobitz II, and eventually to a third-degree AV block. Ultimately, only P waves were observed before cardiac activity ceased. This case provides rare documentation of ECG changes during hanging and may offer further insights into the complex pathophysiology of death by hanging.
